# USE OF ANALYTIC METHODS AND MEDICARE DATA IN THE ANALYSES OF DISPARITIES IN AD/ADRD HEALTH OUTCOMES

**DOI:** 10.1093/geroni/igz038.1593

**Published:** 2019-11-08

**Authors:** Igor Akushevich, Arseniy Yashkin, Svetlana Ukraintseva, Anatoliy Yashin

**Affiliations:** 1 Duke University, Durham, North Carolina, United States; 2 Duke University, Durham, NC, North Carolina, United States

## Abstract

We demonstrate how application of analytic approaches developed in demography, biodemography, epidemiology, and population studies to Medicare and Medicare-linked data allows to identify causes and mechanisms of disparities in AD/ADRD. Our studies i) confirmed geographic disparities in AD mortality (e.g., existence of hot spots, West-East gradient) but did not detect them in Medicare data, ii) confirmed racial disparities in AD/ADRD incidence and survival (e.g., higher incidence and better survival in Black population) and demonstrated that they can be partly explained by heterogeneity in diagnosis severity and partly by genetic factors, iii) detected unexpectedly strong effects of systemic hypotension, chronic kidney and liver diseases on the risk of AD/ADRD. We concluded that further progress in understanding of mechanisms and causes of the disparities in AD/ADRD is possible by clarifying the role of cause-of-death coding, the effects of comorbidity and their treatment, dynamics in cognition scores before AD/ADRD diagnosis, and genetic factors.

